# Should HIV testing for all pregnant women continue? Cost-effectiveness of universal antenatal testing compared to focused approaches across high to very low HIV prevalence settings

**DOI:** 10.7448/IAS.19.1.21212

**Published:** 2016-12-14

**Authors:** Naoko Ishikawa, Shona Dalal, Cheryl Johnson, Daniel R Hogan, Takuro Shimbo, Nathan Shaffer, Razia N Pendse, Ying-Ru Lo, Massimo N Ghidinelli, Rachel Baggaley

**Affiliations:** 1 Division of Communicable Diseases, World Health Organization Regional Office for the Western Pacific, Manila, Philippines; 2 Department of HIV/AIDS, World Health Organization, Geneva, Switzerland; 3 Department of Health Statistics and Information Systems, World Health Organization, Geneva, Switzerland; 4 Ota Nishinouchi Hospital, Fukushima, Japan; 5 Communicable Diseases Department, World Health Organization Regional Office for the South-East Asia, New Delhi, India; 6 Communicable Diseases and Health Analysis, Pan American Health Organization, Washington, DC, USA

**Keywords:** HIV, mother-to-child transmission, HIV testing, cost-effectiveness, universal approach, focused approach

## Abstract

**Introduction:**

HIV testing is the entry point for the elimination of mother-to-child transmission of HIV. Decreasing external funding for the HIV response in some low- and middle-income countries has triggered the question of whether a focused approach to HIV testing targeting pregnant women in high-burden areas should be considered. This study aimed at determining and comparing the cost-effectiveness of universal and focused HIV testing approaches for pregnant women across high to very low HIV prevalence settings.

**Methods:**

We conducted a modelling analysis on health and cost outcomes of HIV testing for pregnant women using four country-based case scenarios (Namibia, Kenya, Haiti and Viet Nam) to illustrate high, intermediate, low and very low HIV prevalence settings. We used subnational prevalence data to divide each country into high-, medium- and low-burden areas, and modelled different antenatal and testing coverage in each.

**Results:**

When HIV testing services were only focused in high-burden areas within a country, mother-to-child transmission rates remained high ranging from 18 to 23%, resulting in a 25 to 69% increase in new paediatric HIV infections and increased future treatment costs for children. Universal HIV testing was found to be dominant (i.e. more QALYs gained with less cost) compared to focused approaches in the Namibia, Kenya and Haiti scenarios. The universal approach was also very cost-effective compared to focused approaches, with $ 125 per quality-adjusted life years gained in the Viet Nam-based scenario of very low HIV prevalence. Sensitivity analysis further supported the findings.

**Conclusions:**

Universal approach to antenatal HIV testing achieves the best health outcomes and is cost-saving or cost-effective in the long term across the range of HIV prevalence settings. It is further a prerequisite for quality maternal and child healthcare and for the elimination of mother-to-child transmission of HIV.

## Introduction

The prevention of mother-to-child transmission (PMTCT) of HIV by providing antiretroviral therapy (ART) to HIV-positive pregnant women is a highly effective intervention to prevent new HIV infections among infants. Globally, an estimated 220,000 children were newly infected with HIV in 2014, a decline of 58% from what was estimated for 2000 [1]. Several countries are moving towards the elimination of mother-to-child transmission (MTCT) of HIV.

HIV testing is the entry point to PMTCT. The elimination of MTCT requires high coverage for both HIV testing and ART; global criteria for elimination include a ≥95% coverage of HIV testing among pregnant women and ≥90% of ART coverage of HIV-positive pregnant women [2]. There has been a substantial scale-up in HIV testing in antenatal care (ANC) settings; yet, in 2014 only about half of pregnant women in low- and middle-income countries received HIV testing services [3].

Resources invested in the HIV response in low- and middle-income countries reached $ 21.7 billion in 2015, of which 57% came from domestic sources [1]. Calls for sustainable financing and effective use of resources are stronger than ever [4–6]. Flat and decreasing external funding for HIV in low- and middle-income countries has triggered a question of whether a focused approach to HIV testing targeting pregnant women in high-burden areas should be considered as a more cost-effective alternative to universal testing. This is particularly a pressing question for countries with limited domestic resources and heavy reliance on external funding. While previous studies confirmed the cost-effectiveness of PMTCT services across high- to low-income settings [7–13], little has been examined on the cost-effectiveness of a focused approach.

This study aimed at examining and comparing the cost-effectiveness of universal and focused approaches in providing HIV testing services for pregnant women in ANC settings across a spectrum of HIV prevalences. This study was conducted as part of the development of the WHO consolidated guidelines on HIV testing services 2015 [14].

## Methods

We conducted a modelling analysis on health and cost outcomes of HIV testing for PMTCT of HIV. We used the Costing Tool for Elimination Initiative, which was developed to estimate the health and cost impact of PMTCT services at national or sub-national levels and was used in Zambia, the Lao People's Democratic Republic and several countries in the Region of the Americas [7,15]. This is an Excel-based tool, publicly available in English, Spanish and French. The details about the tool are also discussed elsewhere [16]. Probabilities of MTCT were based on the estimates provided by the UNAIDS Reference Group on Estimates, Modelling and Projections, which consider both peripartum and postnatal transmission during the breastfeeding period. The analysis was conducted from a health systems perspective, consistent with those of Ministry of Health.

### Country-based case scenarios

We developed four country-based scenarios to illustrate high, intermediate, low and very low national HIV prevalence settings based on published epidemiological data and reports [17–25]. Namibia (with a national HIV prevalence of 17% among females aged 15 to 49 years), Kenya (7%), Haiti (3%) and Viet Nam (with HIV prevalence of 0.1% among ANC attendees) were selected according to their prevalence levels and availability of sub-national demographic and epidemiological data. Each country was divided into high-, medium- and low-burden areas based on their sub-national HIV prevalence. We used HIV prevalence among pregnant women where available; otherwise, the prevalence among women aged 15 to 49 was used to determine the HIV burden in sub-national areas. The summary of demographic, epidemiological and programmatic data used to develop these country-based cases and their sources are presented in Table 1.

**Table 1:** Model input

	Namibia (high)			Kenya (intermediate)			Haiti (low)			Viet Nam (very low)			Reference
**Epidemiological data**													
National HIV prevalence among women aged 15–49 years		17%			7%			3%			0.1%*		
Sub-national HIV burden (prevalence)	High (>20%)	Medium (10–20%)	Low (<10%)	High (>10%)	Medium (5–10%)	Low (<5%)	High (>3%)	Medium (2–3%)	Low (<2%)	High (>0.2%)	Medium (0.1–0.2%)	Low (<0.1%)	
Estimated proportion of women aged 15–49 years reside in the area	37%	52%	11%	14%	60%	26%	26%	51%	22%	17%	41%	41%	
Estimated proportion of HIV-positive women reside in the area	49%	46%	5%	37%	48%	16%	34%	40%	15%	36%	48%	16%	[17–25]
**PMTCT services**													
ANC coverage (at least once)		97%			92%			90%			94%		
HIV testing at ANC		81%			92%			61%			72%		
ART for HIV-positive pregnant women	69% (85% among those tested positive)				71%		57% (93% among those tested positive)				65%		
**Cost (in USD)**													
Antiretroviral drugs													
Maternal ART (14 weeks of pregnancy to 12 months postnatal)		208											
Paediatric ART (annual cost)													[34]
ABC+3TC+LPV/r (0–3 years old)		258											[33]
ABC+3TC+EFV (3–10 years old)		182											
TDF/3TC/EFV (>10 years old)		136											
Laboratory test													
HIV rapid test (per test)		0.73											[29–32]
CD4 (per test)		5.56											
Viral load (per test)		21.56											
Early infant diagnosis (per test)		8.76											
Laboratory monitoring (paediatric HIV) per year		32.86											
Health services													
Clinic with beds (per visit)		7.59			1.39			1.55			1.90		[35]
Primary level hospital (per visit)		8.65			1.59			1.77			2.17		
**GDP per capita**		5589			1358			824			2052		[39]
**Quality-adjusted life years (QALYs)**													
QALYs gained per infant infection averted		20											[36,37]

ABC, abacavir; ANC, antenatal care; ART, antiretroviral therapy; CD4, T–lymphocyte cell bearing CD4 receptor; EFV, efavirenz; GDP, gross domestic product; LPV/r, lopinavir/ritonavir; TDF, tenofovir disoproxil fumarate; 3TC, lamivudine;

* among ANC attendees.

**Figure 1:**
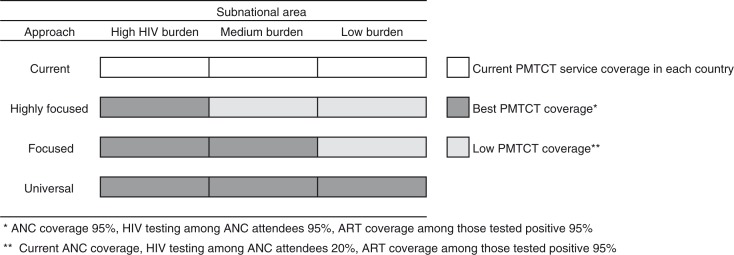
Approaches examined in each country scenario.

The model started with an annual cohort of pregnant women in each country. The base case analysis used the data of current PMTCT service coverage presented in Table 1 as the current approach, followed by the analysis of three different approaches of HIV testing among pregnant women, namely, a highly focused approach, a focused approach and a universal approach. Details of these approaches are summarized in Figure 1. First, in the highly focused approach, we assumed that in high-burden areas 95% of pregnant women attended ANC at least once, 95% of ANC attendees received HIV testing and 95% of those who tested HIV positive received ART (i.e. the best PMTCT coverage). In medium- and low-burden areas, we assumed that only 20% of ANC attendees received HIV testing (i.e. low PMTCT coverage, which consisted of the country's current ANC coverage, 20% HIV testing coverage among ANC attendees, and 95% ART coverage among those who tested HIV positive). Second, in the focused approach, both high- and medium-burden areas had the best PMTCT coverage and the low-burden area had low PMTCT coverage. Finally, in the universal approach, we assumed that all areas had the best PMTCT coverage.

### Virtual country scenario

A virtual country case scenario was developed to conduct sensitivity analysis to further examine the impact of the focused approach beyond the four country-based case scenarios. The levels of national HIV prevalence and other key parameters were varied. It was assumed that the number of annual live births was 1,000,000. The country was divided into high- and low-burden areas, and women aged 15 to 49 were assumed to live equally distributed among them. We also assumed that 70% of all HIV-positive women lived in the high-burden areas. Based on the World Health Statistics 2014 and the Global Update on the Health Sector Response to HIV 2014, we assumed the current coverage of PMTCT services as follows: ANC 75%, HIV testing among pregnant women 74% and ART among HIV-positive pregnant women 67% [17,26]. The summary of assumptions used for the virtual country scenario and the approaches analyzed are included in Supplementary Table 1.

We examined 1) the current approach (current PMTCT coverage for all areas), 2) a focused approach with the best PMTCT coverage for high-burden areas and low coverage for low-burden areas and 3) a universal approach with the best PMTCT coverage for all areas. HIV prevalence was varied between 20 and 0.0005%. The sensitivity analysis also varied the following parameters: proportion of HIV-positive women living in high-burden area, cost of HIV testing and paediatric treatment cost for infected children.

### Assumptions and model inputs

We assumed that pregnant women were tested for HIV at their first ANC visit. We applied two WHO-recommended testing strategies: one for high-prevalence settings (≥5%) and the other for low-prevalence settings (<5%). Retesting of all HIV-negative pregnant women for high-prevalence settings, as well as all HIV-positive pregnant women before initiating ART, was also factored in as per WHO recommendations [14]. We assumed that women identified as HIV positive received ART regardless of WHO clinical stage and at any CD4 cell count, all women breastfed for 12 months and exposed children were followed up through the age of 18 months based on WHO recommendations [27,28]. The cost of future paediatric HIV treatment for 20 years was estimated assuming infected children receive ART based on the regimens recommended by WHO at the time of this analyses [27].

Unit costs for HIV rapid testing, early infant diagnosis, CD4 and viral load monitoring were estimated based on the unit costs from WHO and other sources [29–32]. The cost of antiretroviral (ARV) drugs was based on the WHO report [33] and the Clinton Health Access Initiative ARV Ceiling Price List [34]. Health service costs for each PMTCT follow-up visit were based on WHO Choosing Interventions that are Cost Effective [35]. We assumed that 20 quality-adjusted life years ( QALYs) would be gained by averting a new paediatric HIV infection [36,37]. The main model inputs are summarized in Table 1.

### Main outcomes

We estimated the number of HIV-positive pregnant women identified, the number of new paediatric HIV infections, the number of paediatric infections averted and the MTCT rate per annual cohort of pregnant women. We also estimated the total cost of HIV testing, PMTCT services and the future cost of paediatric HIV treatment. Costs were discounted at 3% annually. We then compared these outcomes by different testing approaches and performed cost-effectiveness analysis. The incremental cost-effectiveness ratios (ICERs) as the incremental cost per paediatric infection averted or per QALY gained in comparisons with the next least-expensive alternative approach were estimated. Following WHO guidance, we considered approaches with ICERs below the gross domestic product (GDP) per capita to be “very cost-effective” and that below three times of GDP to be “cost-effective” [38].

## Results

### Country-based case analysis

Under the highly focused approach, the estimated proportions of pregnant women living in the focused areas ranged from 14 to 37%, which was expected to capture 34 to 49% of HIV-positive women in these country scenarios. For the focused approach, the estimated proportions of pregnant women and women living with HIV in the focused areas were between 58 and 89% and 74 and 95%, respectively.

**Figure 2:**
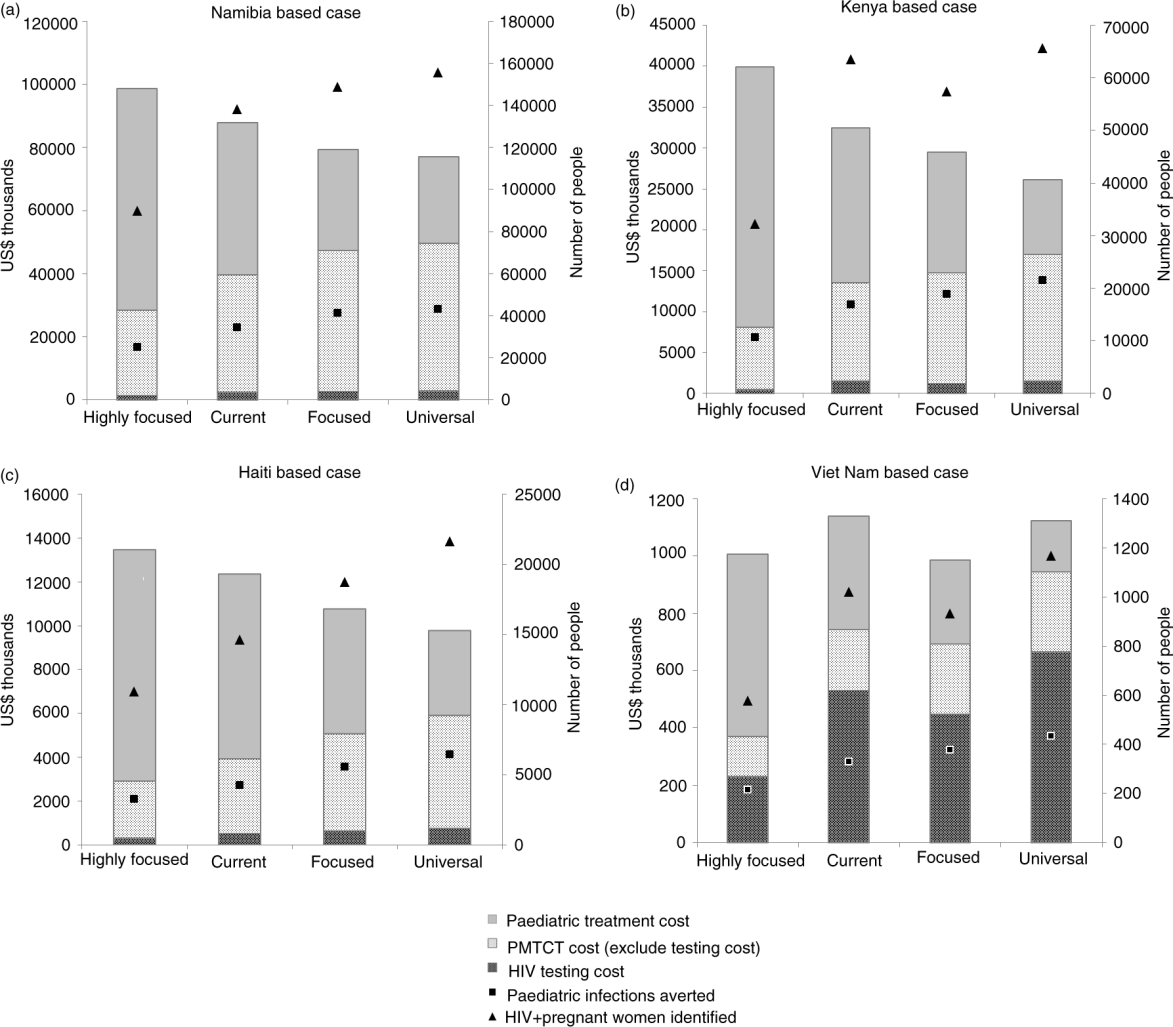
Summary health and cost outcomes of different approaches in four country-based scenarios (per 1,000,000 pregnant women).

The summary results are presented in Table 2 and Figure 2. Under the current approach, MTCT rates were estimated between 12 and 17% in the four country cases. Health outcomes including the number of infections averted, MTCT rate and QALYs gained were superior for the focused and the universal approaches compared with the current approach. In contrast, the highly focused approach resulted in the poorest health outcomes, including higher MTCT rates between 18 and 23%. Higher paediatric treatment costs were also observed under the highly focused approach in all scenarios, for example, over a 60% increase in the Kenya- and Viet Nam-based cases compared with the current approach.

**Table 2:** Health outcomes, costs and cost-effectiveness of different HIV testing approaches in four country-based scenarios (per 1,000,000 pregnant women)

		Health outcomes					Costs (USD thousands)				ICER^a^
				
Country-based case (HIV prevalence among pregnant women)	Approach	MTCT Rate (%)	Number of HIV+ women identified	Number of new paediatric infections	Number paediatric infections averted	Quality-adjusted life years (QALYs) gained	PMTCT (HIV test, ARVs, and health services)	Paediatric treatment (20 years)	Total cost (PMTCT including HIV testing & paediatric treatment)	Cost saved^b^	USD per QALY saved
Namibia (17%)	Universal	7	155,765	11,740	43,102	862,040	49,604	27,654	77,258	128,616	
	Focused	8	148,803	13,667	41,175	823,508	47,324	32,152	79,476	126,398	Dominated^c^
	Current	12	138,221	20,621	34,221	684,426	39,651	48,385	88,035	117,838	Dominated
	Highly focused	18	89,710	30,019	24,824	496,475	28,446	70,322	98,768	107,106	Dominated
Kenya (7%)	Universal	6	65,658	4350	21,551	431,016	17,055	9121	26,175	60,448	
	Focused	10	57,366	7053	18,848	376,953	14,785	14,751	29,536	57,087	Dominated
	Current	13	63,584	9041	16,860	337,200	13,610	18,891	32,501	54,122	Dominated
	Highly focused	22	32,175	15,266	10,635	212,703	8096	31,856	39,952	46,671	Dominated
Haiti (3%)	Universal	8	21,646	1838	6434	128,689	5915	3864	9778	17,976	
	Focused	11	18,731	2705	5568	111,357	5089	5674	10,763	16,992	Dominated
	Current	17	14,635	4014	4259	85,178	3926	8408	12,334	15,420	Dominated
	Highly focused	21	10,923	5026	3247	64,937	2921	10,522	13,444	14,310	Dominated
Viet Nam (0.1%)	Focused	11	1020	139	378	7565	694	292	987	759	
	Highly focused	23	577	303	214	4284	370	638	1008	738	Dominated
	Universal	6	1168	84	433	8663	947	177	1123	622	125
	Current	15	932	188	328	6569	744	397	1141	605	Dominated

a Based on total cost (i.e. PMTCT cost including HIV testing+paediatric treatment cost);

b cost saved=(total costs of no PMTCT intervention) – (total costs of selected approach), where total cost includes HIV testing costs+PMTCT cost+paediatric treatment costs for 20 years;

c an approach that is more expensive and less effective than an alternative approach. MTCT, mother-to-child transmission; PMTCT, prevention of mother-to-child transmission.

The provision of PMTCT services was found to be cost-saving in all country cases and approaches when compared with no PMTCT intervention. When both PMTCT costs and the future paediatric treatment costs were considered, the universal approach was dominant (i.e. more QALYs gained with less cost) compared with the focused and highly focused approaches in the Namibia-, Kenya- and Haiti-based country cases by averting more infections with lower total costs. In Viet Nam-based scenario, the universal approach averted more HIV infections with relatively similar total costs compared with the focused and highly focused approaches; and the universal approach was found to be very cost-effective compared with the focused approach, with an additional $ 125 per QALY gained. When only PMTCT costs were considered, the cost per QALY gained by averting new HIV infections among infants still remained below the GDP per capita in all country scenarios including Viet Nam (Supplementary Table 2).

### Virtual country scenario

We conducted sensitivity analysis using virtual country case scenarios, by varying levels of HIV prevalence between 20 and 0.0005% as well as other key parameters as shown in Supplementary Table 1. Under the assumption that 70% of women living with HIV reside in high-burden areas, the focused approach was found to result in poor outcomes including higher MTCT rates and increased number of new paediatric HIV infections compared with the universal approach. These findings remained constant across the different levels of HIV prevalence.

Both the universal and focused approaches were found to be cost-saving compared with no intervention, even when HIV prevalence was as low as 0.08%. This finding was robust even with higher health service costs and different proportions of HIV-positive women living in high-burden areas. When the cost of paediatric treatment was varied and increased to $ 300 per person per year, all approaches were found to be consistently cost-saving, including with HIV prevalences as low as 0.05%. When the cost of paediatric treatment was increased to $ 1000 per person per year, the approaches were still cost-saving at HIV prevalence of 0.02%.

We calculated the cost per QALY gained based on PMTCT programme costs and future paediatric treatment costs. Our analysis found that the universal approach with the best PMTCT coverage was dominant compared with the focused approach with an HIV prevalence of down to 0.25%. The ICER of the universal approach compared with the focused approach was $ 101 per QALY gained at an HIV prevalence of 0.1%, which is well below the GDP per capita in low- and middle-income countries. When prevalence was reduced to 0.01%, the ICER was $ 1673, which was still highly cost-effective for a low HIV prevalence country like Viet Nam with a GDP per capita of $ 2052 [39]. When compared with the current approach, the universal approach was still cost-effective at an HIV prevalence of 0.005% with GDP per capita of $ 1000.

**Figure 3:**
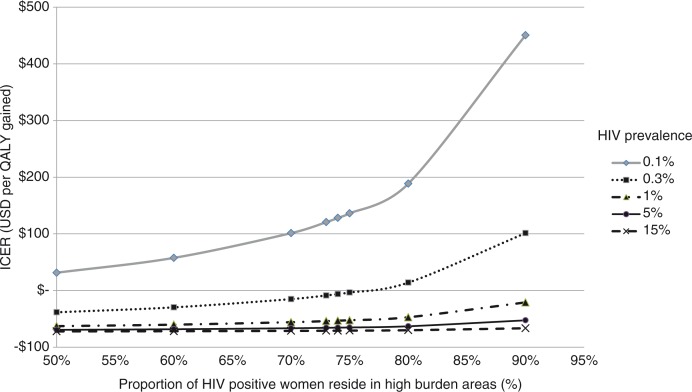
Sensitivity analysis on the impact of HIV prevalence and proportion of HIV-positive pregnant women residing in high-burden areas (universal vs. focused approach).

To examine the impact of sub-national prevalence, the proportion of HIV-positive women in the high-burden areas was varied between 50 and 90% (Figure 3). The universal approach was dominant with an HIV prevalence of 0.3%, with 75% of HIV-positive women living in high-burden areas. Under an HIV prevalence of 0.1%, the ICER increased to $ 451 per QALY gained when 90% of HIV-positive women lived in high-burden areas, which was still well below GDP per capita of most low-income countries [39], and thus regarded as very cost-effective. With a prevalence of 0.01% and 90% of HIV-positive women residing in high-burden areas, the ICER was estimated at $ 5165 per QALY gained, which could still be cost-effective.

## Discussion

We analyzed the cost-effectiveness of universal and focused approaches to antenatal HIV testing across different HIV prevalence settings. We found that the universal approach identifies most HIV-positive mothers, minimizes the number of infections among infants and saves resources by averting future paediatric treatment costs. Although it requires more upfront costs, the universal approach leads to better health outcomes and is cost-saving or cost-effective in the long term.

To our knowledge, this is the first study to compare the health and cost impacts of different HIV testing approaches for pregnant women using country-based scenarios across different HIV epidemiological profiles. This is particularly relevant when considering elimination of mother-to-child HIV transmission in a context of limited resources and optimized investment.

The cost-effectiveness of PMTCT services has been examined in several countries in the past. Early research, mostly conducted in high-income countries in the late 1990s and the early 2000s, analyzed the cost-effectiveness of universal testing in ANC settings. Studies from the United Kingdom and Australia addressed the provision of universal antenatal HIV screening and its cost-effectiveness in settings of very low HIV prevalence [8,9]. Both studies concluded that universal HIV testing would be cost-effective even with very low HIV prevalence and recommended its inclusion in routine ANC. Currently, HIV screening is considered as a component of standard antenatal care in many countries [40–44].

Recent studies on PMTCT in sub-Saharan Africa, where HIV prevalence is high and universal HIV testing for pregnant women is strongly recommended, analyzed the cost-effectiveness of different options for PMTCT services and concluded that they were cost-effective [7,10–12]. An analysis of PMTCT programmes in New York State between 1998 and 2013 found that every $ 1 invested in PMTCT, $ 4 has been saved in HIV treatment costs; and concluded that it justified the allocation of resources for PMTCT [13]. Our analysis supports the evidence from the past studies that PMTCT with universal antenatal HIV testing is a cost-saving and cost-effective intervention even in low HIV prevalence settings. We also found that the universal approach proved to be very comparable with other priority interventions such as early initiation of ART in serodiscordant couples ($ 590 per life-year saved in South Africa; $ 442 per QALY gained in India) in terms of its cost-effectiveness [45,46].

In the current environment of flat-lined and decreasing HIV funding [1], there has been a call by external donors and partners for more efficient allocation of resources, including the focused and prioritized HIV testing approaches targeting high HIV burden areas [5]. Our comparison of the universal and focused approaches found that while the focused approach would imply decreased expenditure for HIV testing and PMTCT services in the short term, it would result in higher expenditure in the long term due to a larger number of new paediatric infections. The universal approach was dominant in preventing more paediatric HIV infections with lower total costs even in ANC settings with HIV prevalence as low as 0.25%. The universal approach maintained cost-effectiveness in settings with an HIV prevalence of 0.003% and GDP per capita of $ 2000. Even in a scenario where 90% of HIV-positive women live in high-burden areas, the universal approach was still cost-effective.

The findings of this study should be interpreted taking into consideration the following limitations. We may have underestimated the health impact of interventions since the health benefit for women starting early ART and its impact on HIV transmission among serodiscordant couples were not included in our analysis. The future cost-savings may be underestimated as our model used the low-end cost for paediatric treatment and limited the treatment time horizon to 20 years. In addition, we did not discuss or model the potential additional benefits of bundling HIV testing, with syphilis or hepatitis B testing. We adopted a conservative approach and believe that these limitations would, in fact, further strengthen the argument in favour of the universal approach, as the benefits and the cost-effectiveness of PMTCT interventions on maternal health outcomes and prevention of partner infection are well documented [7,11,47]. We are also aware that our scenario did not take into account the non-breastfeeding population, which may have resulted in overestimation of MTCT rates. It should also be noted that our scenarios are not exhaustive; we are aware of country-specific epidemic situations that may not fit into any of the four cases, including the coexistence of different HIV epidemic situations among certain populations within the same country. It should also be noted that our parameters of “best PMTCT coverage” do not support achievement of the elimination target of <5% MTCT rates, as they only assume an 86% ART coverage among HIV-positive women (as a result of 95% ANC coverage, 95% HIV testing among ANC attendees and 95% ART coverage among those who tested HIV positive). Our scenarios did not take into account potentially lower rates of ANC attendance among women at higher risk of HIV infection, particularly women who inject drugs and young women involved in transactional sex who are often marginalized. Countries with low or variable ANC coverage need to consider additional investments for reaching women and linking them to ANC services, particularly those at higher risk of HIV infection who may not access ANC.

The universal approach is optimal when striving towards the elimination of new paediatric HIV infections. The question to be answered is: should we invest now to prevent new HIV infections or pay later for HIV treatment? Countries with limited resources may face a difficult decision in allocating currently available funds among many competing priorities. For countries facing resource challenges, a focused approach could be applied in the short term as an interim measure and then scale up to a universal approach as resources are identified to provide universal HIV testing for pregnant women. Last but not least, access to HIV testing for all people, including women and children living in non-focused areas who wish to be tested, needs to be ensured.

## Conclusions

Universal approach to antenatal HIV testing achieves the best health outcomes and is cost-saving or cost-effective in the long term across the range of HIV prevalence settings. It is further a prerequisite for quality maternal and child healthcare and for the elimination of MTCT of HIV.

## Supplementary Material

Should HIV testing for all pregnant women continue? Cost-effectiveness of universal antenatal testing compared to focused approaches across high to very low HIV prevalence settings
