# Supplementary material for: Should HIV testing for all pregnant women continue? Cost-effectiveness of universal antenatal testing compared to focused approaches across high to very low HIV prevalence settings
Source: J Int AIDS Soc. 2016 Dec 14;19(1):21212. doi: 10.7448/IAS.19.1.21212 (PMC5159683; doi:10.7448/IAS.19.1.21212)

Supplementary table 1. Health outcomes, costs and cost-effectiveness of different HIV testing approaches in four country-based cases (per 1,000,000 pregnant women)

		Health Outcomes					Costs (US$ thousands)					Incremental cost-effectiveness ratio (US$) based on PMTCT cost [2]	
Country-based case (HIV prevalence among pregnant women)	Approach	MTCT Rate	Number of HIV+ women identified	Number of new paediatric infections	Number paediatric infections averted	Quality-adjusted life years (QALYs) gained	HIV testing (test kit + services)	PMTCT (HIV test, ARVs, and health services)	Paediatric treatment (20 years)	Total cost (PMTCT including HIV testing + paediatric treatment)	Cost saved [1]	cost per infection averted	cost per QALY gained
Namibia (17%)	Highly focused	18%	89,710	30,019	24,824	496,475	1,527	28,446	70,322	98,768	107,106		
	Current	12%	138,221	20,621	34,221	684,426	2,541	39,651	48,385	88,035	117,838	weakly dominated	weakly dominated
	Focused	8%	148,803	13,667	41,175	823,508	2,673	47,324	32,152	79,476	126,398	1,154	58
	Universal	7%	155,765	11,740	43,102	862,040	2,863	49,604	27,654	77,258	128,616	1,183	59
Kenya (7%)	Highly focused	22%	32,175	15,266	10,635	212,703	504	8,096	31,856	39,952	46,671		
	Current	13%	63,584	9,041	16,860	337,200	1,513	13,610	18,891	32,501	54,122	weakly dominated	weakly dominated
	Focused	10%	57,366	7,053	18,848	376,953	1,249	14,785	14,751	29,536	57,087	814	41
	Universal	6%	65,658	4,350	21,551	431,016	1,562	17,055	9,121	26,175	60,448	840	42
Haiti (3%)	Highly focused	21%	10,923	5,026	3,247	64,937	326	2,921	10,522	13,444	14,310		
	Current	17%	14,635	4,014	4,259	85,178	522	3,926	8,408	12,334	15,420	weakly dominated	weakly dominated
	Focused	11%	18,731	2,705	5,568	111,357	638	5,089	5,674	10,763	16,992	934	47
	Universal	8%	21,646	1,838	6,434	128,689	771	5,915	3,864	9,778	17,976	953	48
Viet Nam (0.1%)	Highly focused	23%	577	303	214	4,284	231	370	638	1,008	738		
	Focused	11%	1,020	139	378	7,565	448	694	292	987	759	1,977	99
	Current	15%	932	188	328	6,569	531	744	397	1,141	605	dominated	dominated
	Universal	6%	1,168	84	433	8,663	665	947	177	1,123	622	4,601	230

[1] as compared to no PMTCT interventions

[2] based on PMTCT cost only, does not include paediatric treatment cost

Supplementary table 2. Virtual country scenario

Country scenario	Annual live births: 1 000 000	
	Population: 50% reside in high HIV burden area and 50% in low HIV burden area	
	Proportion of HIV-positive women reside in high burden area: 70%	
	PMTCT services: ANC coverage 75%, HIV testing coverage 74% among all pregnant women, ART coverage among HIV-infected pregnant women 67%	
	Unit cost: same as 4 country-based case scenarios	
Key parameters	HIV prevalence	0.0005% - 20%
	HIV-positive women in high burden area	50% - 90%
	HIV testing cost	1 US$ - 6 US$/test
	Paediatric treatment cost (annual)	300 US$ - 1000 US$/person

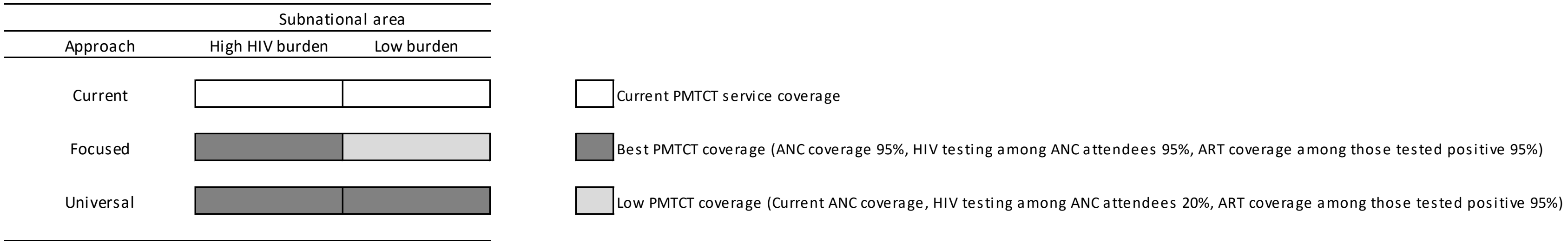

Supplement: Should HIV testing for all pregnant women continue? Cost-effectiveness of universal antenatal testing compared to focused approaches across high to very low HIV prevalence settings [file JIAS-19-21212-s001.pdf]
